# PRESIDENTIAL SYMPOSIUM: RESILIENCE IN OLDER ADULTS: A MULTIDIMENSIONAL PERSPECTIVE

**DOI:** 10.1093/geroni/igae098.1677

**Published:** 2024-12-31

**Authors:** Mo-kyung Sin, Pamela Cacchione

**Affiliations:** Chair; Discussant

## Abstract

In alignment with the theme of this year’s conference “The Fortitude Factor”, this symposium focuses on multidimensional perspectives of resilience in older adults. Resilience in older age is the ability to stand up to adversity and to bounce back to a state of equilibrium following individual adverse events. Maintaining physical, psychological, and cognitive resilience is an integral component for quality life and well-being in older adults. Whereas, conceptualizing and properly operationalizing resilience is essential for advancing research to achieve well-being of older adults. Our first presenter will discuss the psychometrics of the physical resilience scale in older adults living with dementia. Our second presenter will discuss psychological evaluation of resilience and provide insights gained from her research on the protective effects of psychological resilience across stressors and outcomes. Our last presenter will share factors associated with cognitive reserve from the BIOCARD study. Given the association between resilience and wellbeing in older adults, this HS section Presidential Symposium is sure to challenge and enhance our thinking to advance science.

